# Defect-engineered competition between exciton annihilation and trapping in MOCVD WS_2_

**DOI:** 10.1039/d5sc07343j

**Published:** 2025-11-14

**Authors:** Ruofei Zheng, Leon Daniel, Dedi Sutarma, Christian Viernes, Yingfang Ding, Tobiloba Fabunmi, Gerd Bacher, Michael Heuken, Holger Kalisch, Andrei Vescan, Peter Kratzer, Marika Schleberger, Germán Sciaini

**Affiliations:** a Department of Chemistry, University of Waterloo Waterloo Ontario N2L 3G1 Canada gsciaini@uwaterloo.ca; b Fakultät für Physik and CENIDE, University of Duisburg-Essen Duisburg 47057 Germany marika.schleberger@uni-due.de; c Compound Semiconductor Technology, RWTH Aachen University Aachen 52074 Germany; d Werkstoffe der Elektrotechnik and CENIDE, University of Duisburg-Essen Duisburg 47057 Germany; e AIXTRON SE Herzogenrath 52134 Germany

## Abstract

Exciton dynamics critically influence the optoelectronic performance of two-dimensional transition metal dichalcogenides (TMDCs). In large-scale WS_2_ monolayers grown *via* metal–organic chemical vapor deposition (MOCVD), intrinsic sulfur vacancies introduce in-gap states that promote nonradiative recombination through defect trapping (DT). Under elevated excitation conditions, the decay behaviour changes as exciton–exciton annihilation (EEA) emerges as a competing nonradiative process. To investigate these mechanisms across excitation regimes, we combine steady-state quantum efficiency measurements with femtosecond broadband transient absorption spectroscopy on samples with varying defect concentrations. These complementary measurements provide an unprecedented quantitative disentanglement of these decay pathways, a level of analysis not previously reported for MOCVD-grown monolayer WS_2_. The induced defect states are partially occupied, as first revealed by sub-bandgap excitation, and variations in defect density exert a pronounced influence on the photo-induced band renormalization. After establishing these DT-specific properties, we apply a rate-equation model including both DT and EEA to extract constants of 0.02 cm^2^ s^−1^ and 0.1 cm^2^ s^−1^, followed by an in-depth exploration of their fundamentally diffusion-limited behaviour. The competition between DT and EEA can be set by a critical defect-to-exciton density ratio (≈3.5), which serves as the threshold for EEA activation. Moreover, at high exciton densities, defect saturation suppresses DT, reshaping the decay landscape. Overall, our findings provide detailed insights into defect-modulated exciton decay mechanisms and establish a quantitative framework for tailoring the optoelectronic properties of TMDCs *via* controlled defect engineering.

## Abstract 2

Steady-state and ultrafast spectroscopy reveal how controlled defect introduction tunes defect trapping and exciton–exciton annihilation in MOCVD-grown WS_2_, establishing a quantitative framework for diffusion-limited exciton dynamics.

## Graphical abstract

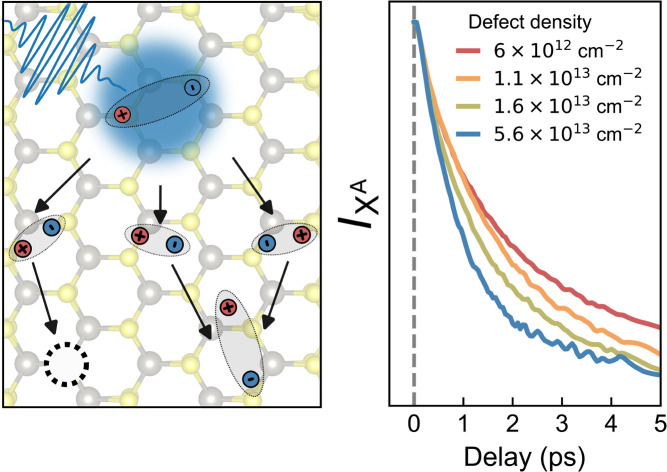

## 1 Introduction

Monolayer transition metal dichalcogenides (TMDCs) have emerged as promising materials for next-generation optoelectronic and valleytronic devices due to their direct bandgap in the visible range, strong light–matter interaction, and high photoluminescence quantum yield.^1,2^ Among TMDCs, tungsten disulfide WS_2_ is particularly attractive owing to its large oscillator strength and favorable excitonic properties,^3–5^ making it a key candidate for light-emitting diodes, photodetectors, and other optoelectronic components.^6,7^

The performance of such devices is intimately linked to the fate of photoexcited excitons. In monolayer WS_2_, excitons decay *via* radiative and nonradiative channels, with the latter often dominating under realistic conditions. Nonradiative losses are primarily governed by two processes: exciton–exciton annihilation (EEA) and defect trapping (DT), the latter of which is mediated by structural imperfections such as sulfur vacancies.^8–12^

Large-area growth methods such as metal–organic chemical vapor deposition (MOCVD) inevitably introduce intrinsic defects,^13–15^ most notably sulfur vacancies, which form in-gap states and act as efficient recombination centers.^12,16,17^ While such defects degrade photoluminescence efficiency, they also offer opportunities for tailoring material properties *via* targeted defect engineering.^18–23^ Moreover, the effectiveness of methods used to enhance the photoluminescence efficiency are strongly correlated with the exciton recombination mechanisms. For instance, reducing trion-related nonradiative recombination through electrostatic gating, or mitigating defect-mediated losses *via* targeted chemical passivation.^24–26^ Understanding the interplay between intrinsic defects and exciton dynamics, particularly on ultrafast timescales, is thus critical for both fundamental insights and device optimisation.

Despite substantial experimental and theoretical work, quantitative analyses of the competing roles of EEA and DT in defective large-scale monolayer WS_2_ remain scarce. In particular, how the dominance of each mechanism varies with excitation conditions and defect concentrations has not been thoroughly discussed. To address this gap, we combine steady-state quantum efficiency measurements with femtosecond transient absorption (TA) spectroscopy to dissect the exciton decay landscape in MOCVD-grown WS_2_. By systematically tuning the defect density *via* controlled ion irradiation, we extract key kinetic parameters and identify a critical defect-to-exciton ratio that governs the onset of EEA. Our findings establish a quantitative framework for exciton recombination in TMDCs, highlighting defect engineering as a powerful means of optoelectronic control, and providing guidance for future strategies to tune quantum efficiency.

## 2 Results and discussion

### 2.1 Defect engineering and steady-state measurements

**Fig. 1:**
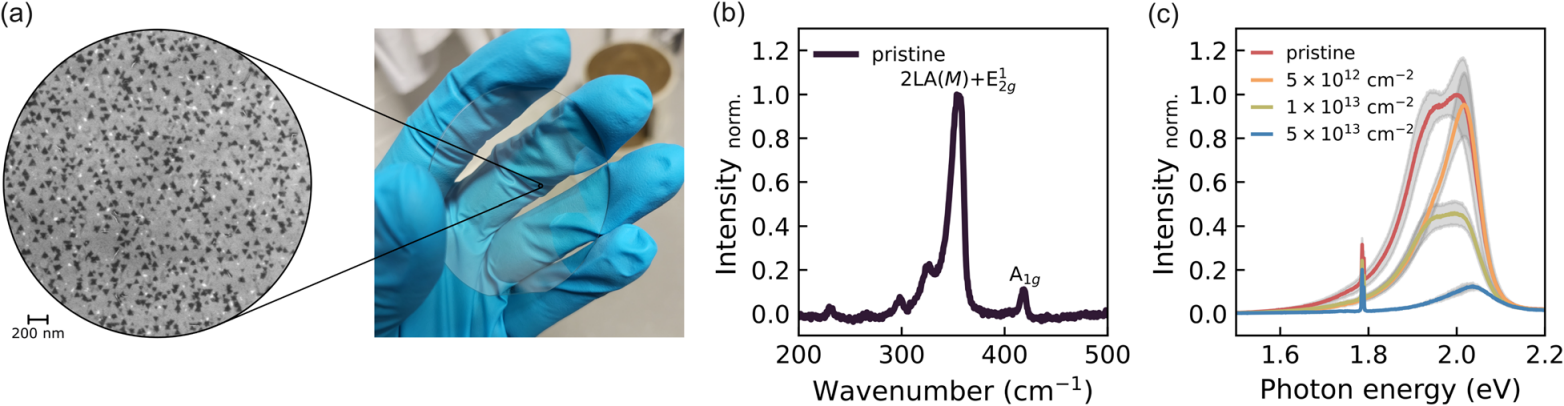
(a) SEM image (left) of wafer-scale monolayer WS_2_ grown *via* MOCVD (right). Dark grey regions indicate bilayer areas, while light grey corresponds to monolayer WS_2_. The white regions indicate uncovered areas. (b) Raman spectrum of the pristine WS_2_ sample, showing prominent A_1*g*_(Γ) and 2LA(*M*) modes. (c) PL spectra of pristine and defective samples. A decrease in intensity and spectral shifts are observed with increasing defect density.

Our WS_2_ monolayers were synthesized *via* metal–organic chemical vapor deposition (MOCVD) on two-inch, double-sided polished sapphire wafers (see Materials and methods for details). This method yields uniform, wafer-scale films with predominantly monolayer coverage and minimal bilayer formation, as illustrated in Fig. 1(a). Scanning electron microscopy (SEM) reveals an overall coverage of ≈98.5%, comprising 81.5% monolayer (light grey) and 17% bilayer regions (dark grey).

Raman and photoluminescence (PL) spectroscopy were employed to characterize the structural and optical properties. The Raman spectrum (Fig. 1(b)) displays prominent A_1*g*_(Γ) and 2LA(*M*) modes, consistent with monolayer WS_2_.^27–29^ The PL spectrum (Fig. 1(c)) exhibits a strong excitonic peak centred at 2 eV, confirming the monolayer character. A secondary feature at lower energy is attributed to trion emission, contributing to a low-energy tail.^30^ Additionally, two weaker peaks near 1.8 eV originate from the sapphire substrate.^31^

The wafer was sectioned into smaller pieces and subjected to argon ion irradiation with energies below 50 eV (see Materials and methods for details). Four sample conditions were investigated: one pristine reference and three irradiated samples with ion fluences of 5 × 10^12^, 1 × 10^13^, and 5 × 10^13^ cm^−2^. A defect creation yield of unity was assumed, consistent with molecular dynamics simulations.^32^ Calculations using the program stopping and range of ions in matter (SRIM) further confirmed negligible sputtering or ion backscattering from the substrate, excluding secondary defect formation.^33^

Ion irradiation predominantly creates single sulfur vacancies in the WS_2_ lattice.^34–36^ A neutral sulfur vacancy introduces two flat in-gap states above the Fermi level and an additional defect level within the valence band.^37,38^ These in-gap states act as efficient nonradiative recombination centers,^16^ leading to a reduction in quantum efficiency (*QE*), as reflected by the decreasing PL intensity with increasing defect density (Fig. 1(c)). No significant variations in the trion-to-exciton ratio are observed across the defect series (Fig. S1). At the highest defect densities, however, a slight blueshift (≈30 meV) appears in the PL spectra, likely due to subtle band renormalization effects induced by sulfur vacancies.

**Fig. 2:**
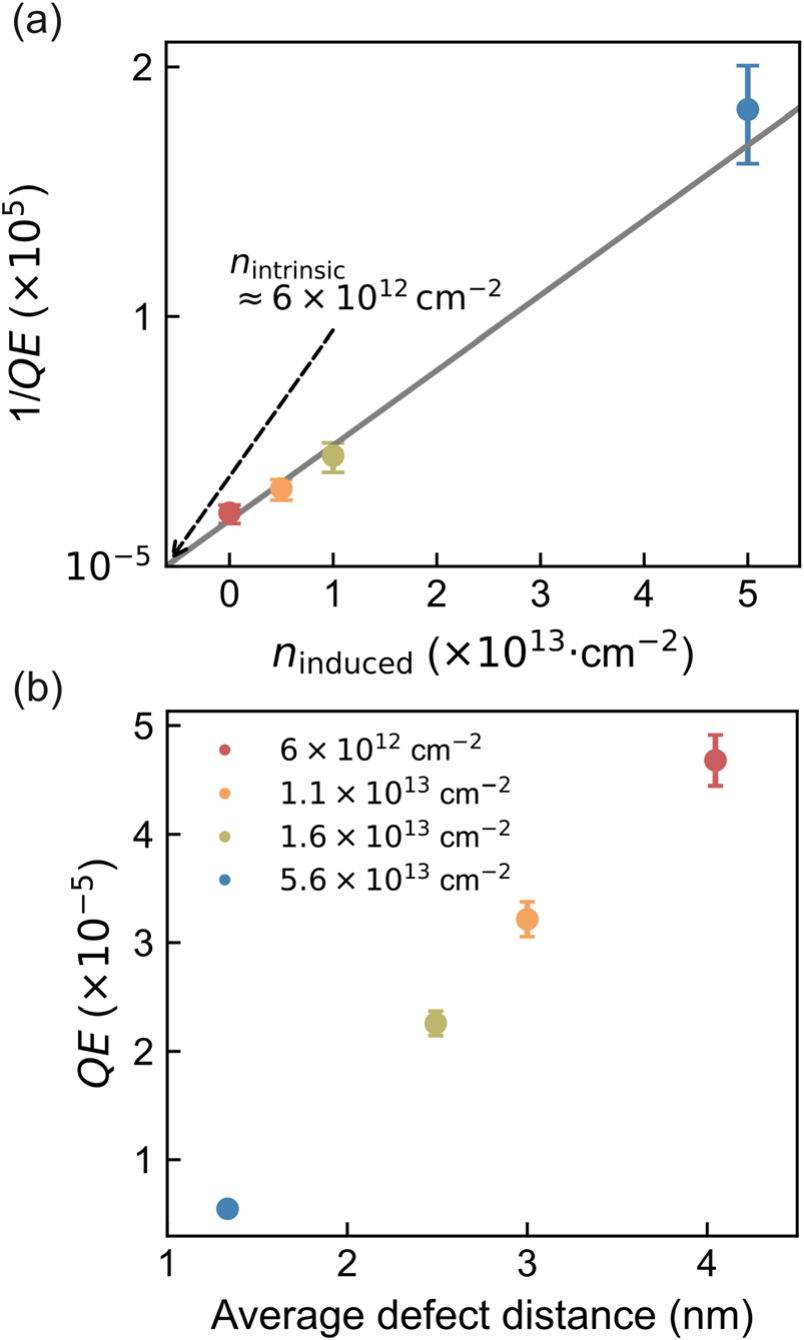
(a) Inverse quantum efficiency (1/*QE*) as a function of induced defect density (*n*_induced_). A decrease in *QE* is observed with increasing defect density, corresponding to an increase in 1/*QE*. The intrinsic defect density (*n*_intrinsic_) in the pristine sample is estimated based on the assumptions applied in the *QE* math model. The colours are consistent with those in the previous PL plot, and the error bars represent the uncertainties in the PL data. (b) *QE* compared with the average defect distance under consideration of the intrinsic defect density, indicating that ion bombardment generates a uniformly distributed set of defect conditions.

To quantify the impact of defects on radiative efficiency, the PL spectra were integrated over the 1.6–2.2 eV range, and the *QE* of pristine and irradiated samples was determined relative to a calibrated reference (see Materials and methods for details). The extracted 1/*QE* values are shown as a function of induced defect density in Fig. 2(a), with error bars reflecting the standard deviation of repeated PL measurements.

To link the observed increase in 1/*QE* to exciton decay dynamics, we introduce a rate-based model that relates *QE* to the competing recombination pathways. Specifically, *QE* can be expressed as:1
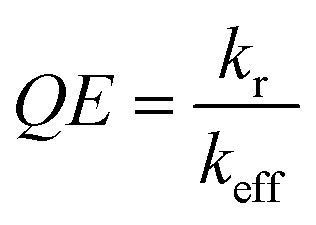
Here, *k*_r_ denotes the radiative recombination rate, while *k*_eff_ = *k*_r_ + *k*_nr_ represents the total recombination rate, comprising both radiative and nonradiative contributions.

Given the low *QE* observed in our samples (≈3.2 × 10^−5^), we approximate *QE* ≈ *k*_r_/*k*_nr_, where *k*_nr_ ≫ *k*_r_. Under these conditions, nonradiative decays dominate, with defect trapping (DT) and exciton–exciton annihilation (EEA) as the two primary decay channels. EEA becomes relevant only at elevated exciton densities, typically above 10^11^ cm^−2^ for defective WS_2_.^39^ In our steady-state measurements, the use of continuous-wave excitation results in low exciton densities, allowing us to neglect EEA. Accordingly, the nonradiative rate simplifies to:2
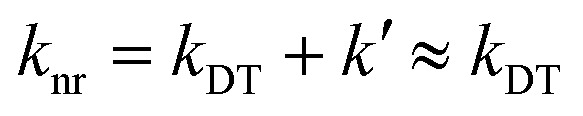
with3
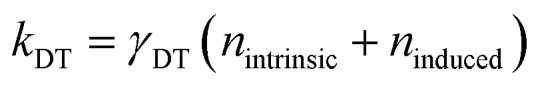


Here, *k*_nr_ reflects the sum of the defect trapping rate *k*_DT_ and additional ultrafast nonradiative processes, collectively denoted as k′. Potential contributions to *k*′ may stem from native defect species not directly modified by ion irradiation, such as tungsten vacancies or defect clusters. Given their comparatively low abundance relative to sulfur vacancies, we treat *k*′ as negligible in this analysis. *k*_DT_ is determined by the product of the trapping rate per defect *γ*_DT_ and the sum of the densities of intrinsic (*n*_intrinsic_) and induced sulfur vacancies (*n*_induced_) generated by ion irradiation.

Based on these assumptions, the intrinsic defect density can be determined by the following equation:4
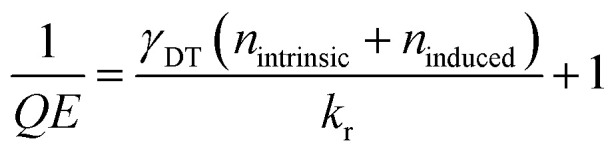


We assume *QE* = 1 in the absence of sulfur vacancies, the dominant nonradiative centers in our system. Based on the linear fit in Fig. 2(a), the intrinsic defect density is estimated to be *n*_intrinsic_ ≈ 6 × 10^12^ cm^−2^, indicating a substantial background of native sulfur vacancies in the pristine sample. Using a reported radiative recombination rate of *k*_r_ ≈ 0.1 ns^−1^ from time-resolved PL measurements (tr-PL) on MOCVD-grown WS_2_,^8,40^ we extract a trapping coefficient of *γ*_DT_ ≈ 3 × 10^−13^ cm^2^ ps^−1^.

This steady-state model forms the basis for comparison with time-resolved measurements, where the interplay between DT and EEA will be examined in detail. Beyond quantifying the intrinsic defect density, the *QE* analysis also provides insight into the spatial distribution of trapping centers. When plotted against the average defect distance, including intrinsic contributions, the data suggest a relatively uniform defect distribution across the irradiated samples (Fig. 2(b)). However, the analysis of DT rate relies on radiative lifetime values derived from tr-PL, which are limited by their temporal resolution. To obtain a more accurate and dynamic picture of defect-mediated exciton decay, we turn to femtosecond transient absorption (TA) spectroscopy in the following section.

### 2.2 Time-resolved measurements

**Fig. 3:**
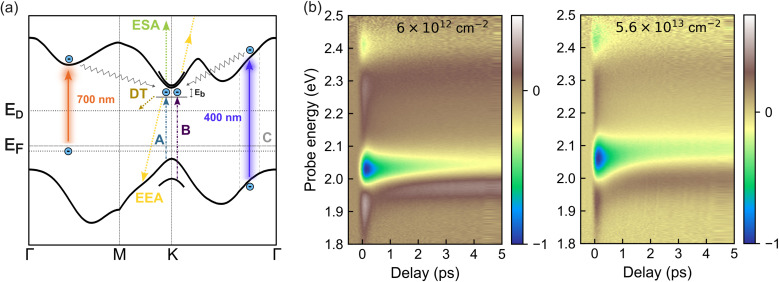
(a) Schematic illustration of exciton dynamics in WS_2_ in the presence of defect states. Under 400 nm excitation, carriers are excited from the valence band to the conduction band near the C exciton region, while 700 nm excitation drives transitions from occupied in-gap defect states to the conduction band. To aid visualization, the 700 nm excitation is depicted as shifted away from the *K* point in momentum space. Generated excitons (after ultrafast relaxation) can decay nonradiatively *via* defect trapping (DT) or exciton–exciton annihilation (EEA). With sufficient photon absorption, carriers can also be excited to higher energy states through excited state absorption (ESA). The band structure, including defect states of a simply charged sulfur vacancy, is shown based on DFT calculations. (b) Colourmaps of the TA spectra for samples with the lowest (left) and highest (right) defect densities. The signals are normalized to their respective minimum signals and plotted as a function of probe energy (eV) and pump–probe delay time (ps).

Time-resolved experiments were conducted as illustrated schematically in Fig. 3(a). Two excitation wavelengths were employed to access distinct electronic transitions. A 400 nm (3.1 eV) pump predominantly excites the high-energy C exciton region, followed by ultrafast relaxation into A and B exciton states within tens of femtoseconds.^41^ The A and B excitons are hallmark features of TMDC monolayers, arising from spin–orbit splitting of the valence band. Both are spin-singlet states, differing only in the relative spin alignment of the electron and hole.^42^ To probe defect-related transitions, additional measurements were performed using sub-bandgap excitation at 700 nm (1.77 eV), targeting in-gap states associated with sulfur vacancies.

Colourmaps of the TA spectra for samples with the lowest and highest defect densities are shown in Fig. 3(b), normalized to their respective minimum signals. The estimated intrinsic sulfur vacancy densities, derived from steady-state analysis, are included for reference. A tailored temporal sampling scheme was employed, using finer steps (down to 50 fs) during the initial picoseconds to capture ultrafast dynamics, and coarser steps at longer delays. Colourmaps for the other two samples are provided in Fig. S2, along with spectral cuts at a delay of 2 ps for all samples.

At this timescale, distinct Pauli blocking features appear at ≈2.05 eV and above 2.4 eV, corresponding to the A- and B-exciton transitions, respectively. These features reflect a reduction in absorption due to state filling by photoexcited excitons.^43^ In addition to Pauli blocking, two positive signal features emerge in the TA spectra. These are attributed to photoinduced excited-state absorption (ESA), in which carriers in the conduction band absorb probe photons to access higher-lying electronic states,^43^ and to photo-induced band renormalization, leading to a spectral shift between pump-off and pump-on conditions.^44^

In the following, we focus on the A-exciton Pauli blocking region to track exciton dynamics as a function of defect density. For consistency, all analyses are based on the absolute value of the differential signal.

### 2.3 Evidence for occupied in-gap states

**Fig. 4:**
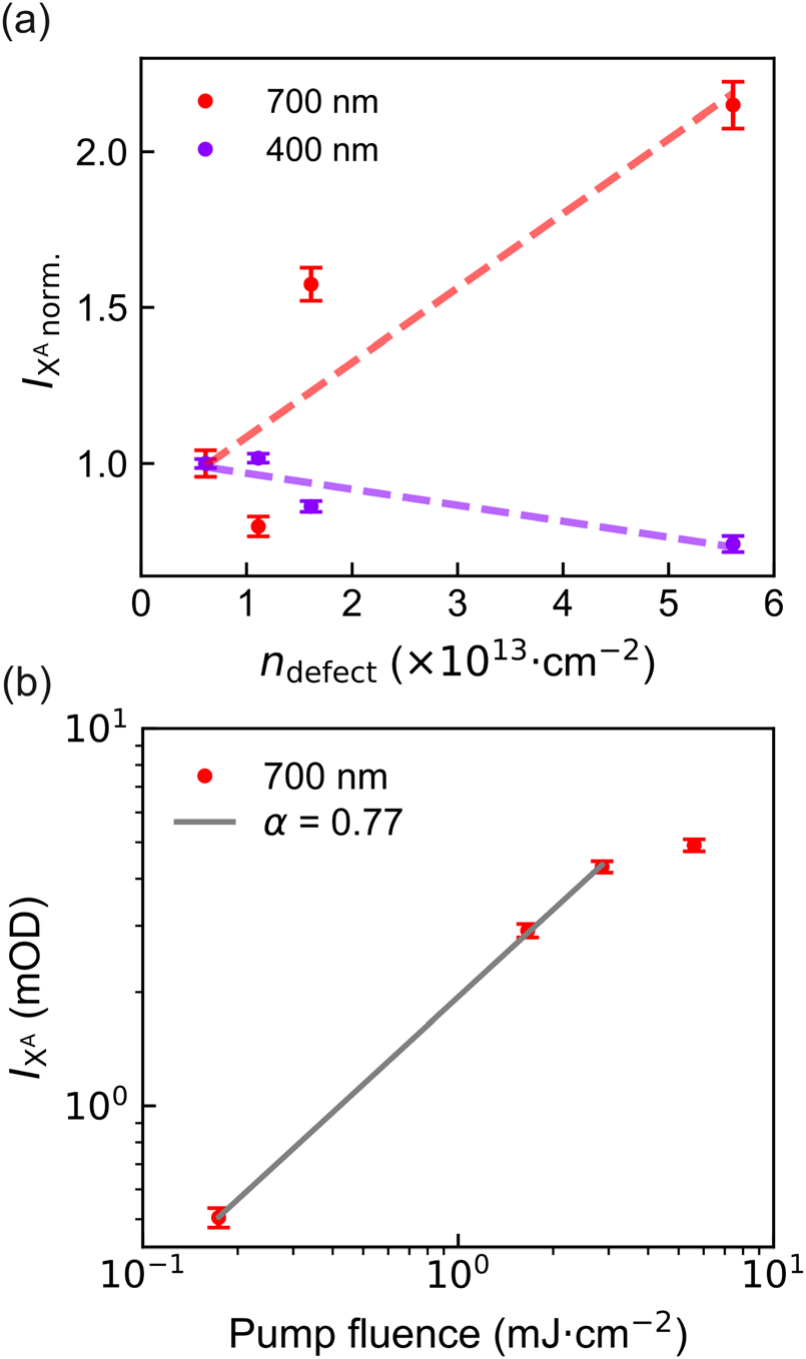
(a) Comparison of A-exciton peak intensities at 0 ps extracted from Gaussian fitting under 400 nm (purple) and 700 nm (red) pump excitation for different defect densities. Intensities are normalized to the least defective sample for both excitation conditions. A slight decrease in intensity with increasing defect density is observed under 400 nm excitation, while 700 nm excitation shows a pronounced increase. The dashed line is solely a visual guide. (b) A-exciton peak intensity as a function of pump fluence under 700 nm excitation, plotted on a log–log scale. The fitted slope of 0.77 indicates that the excitation mechanism is not dominated by two-photon absorption. Error bars in both panels represent uncertainties from Gaussian fitting.

We begin our analysis by examining the signal amplitude at *t* = 0 ps. The absolute A-exciton signal intensity under 400 nm and 700 nm excitation was extracted *via* Gaussian fitting (see Fig. S3) and normalized to the value of the lowest-defect-density sample (Fig. 4(a)). Under 400 nm excitation, a slight decrease in peak intensity is observed with increasing defect density, while sub-bandgap excitation at 700 nm yields a pronounced increase.

For 400 nm excitation, carriers are initially excited into higher-energy states and subsequently relax into the A-exciton manifold.^41^ At higher defect densities, carriers can be increasingly captured by in-gap states before reaching the A-exciton state, resulting in a slightly reduced signal intensity. In contrast, excitation at 700 nm (below the bandgap) is expected to drive transitions from occupied in-gap states into the conduction band. The observed intensity increase with defect density suggests a rising population of such occupied states.

To rule out two-photon absorption as the origin of this signal, we performed fluence-dependent measurements (Fig. 4(b)). The resulting log–log plot exhibits a linear dependence with a slope of 0.77, well below the expected value of 2 for a two-photon process.^45^ This confirms that the excitation originates from one-photon transitions involving occupied in-gap defect states. While such excitation may also generate free carriers that contribute to trion formation, their concentration appears limited. This is evidenced by the substantially lower overall signal amplitude under 700 nm excitation compared to 400 nm, despite the use of significantly higher pump fluence.

These findings are initially surprising, as neutral sulfur vacancies are not expected to introduce occupied in-gap states. To explore this discrepancy, we performed density functional theory (DFT) calculations to assess the electronic structure of charged sulfur vacancies (Fig. S4 and S5). The charge transition level (CTL) from neutral to singly charged *V*_S_− lies at an acceptor level just below the conduction band. Both singly and doubly negatively charged *V*_S_ configurations exhibit multiple occupied in-gap states, consistent with our spectroscopic observations. Experimentally, such negatively charged vacancies have been identified *via* scanning tunneling microscopy (STM), where characteristic contrast was attributed to chalcogen vacancies in distinct charge states.^38^

Defect-induced modifications to the exciton transition energy also become evident when tracking the A-exciton peak position. At *t* = 0 ps, a systematic blueshift is observed with increasing defect density (Fig. S6), in agreement with the PL trend in Fig. 1(c). In addition, a transient blueshift of the exciton resonance is observed over time, attributed to the relaxation of the band structure back to its equilibrium configuration after photoexcitation.^44^ Notably, this relaxation occurs more rapidly in samples with higher defect densities (Fig. S7), suggesting faster carrier localization in the presence of defect states.

### 2.4 Exciton dynamics under strong photoexcitation

**Fig. 5:**
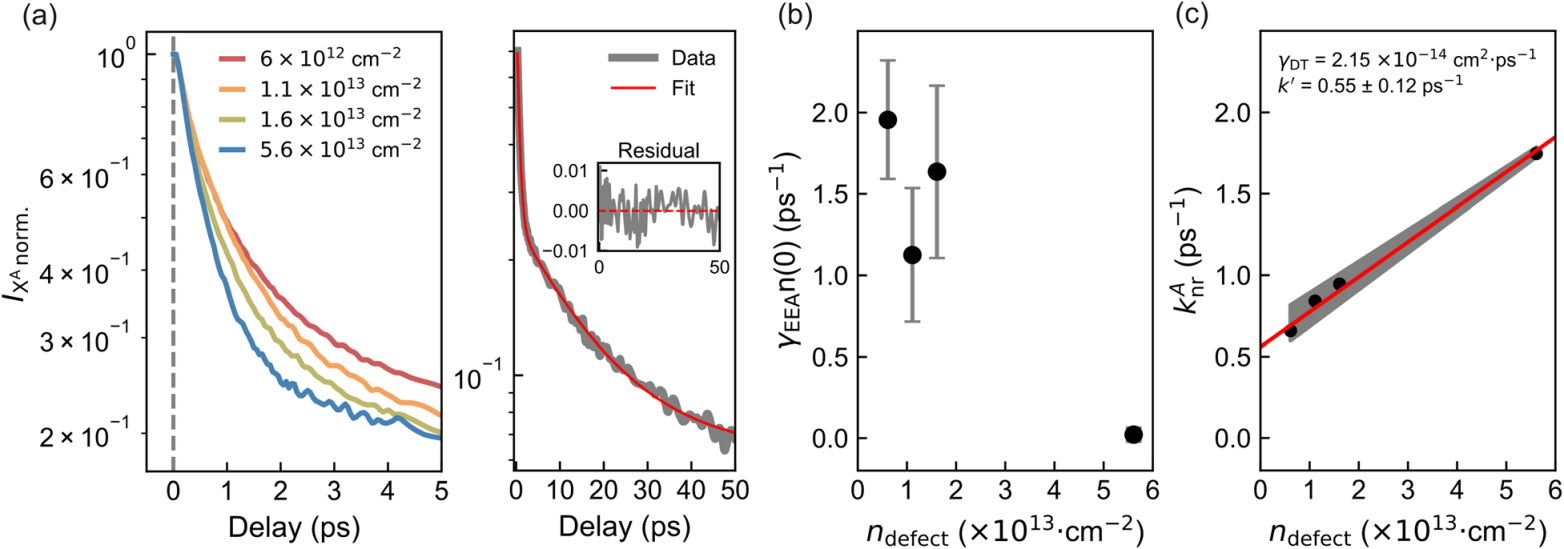
(a) Normalized, shift-corrected A-exciton decay traces of samples with varying defect densities up to 5 ps (left). Representative A-exciton decay fits from 0.3 to 50 ps for the sample with the highest induced defect density (right). Both panels are plotted on a logarithmic scale. The corresponding fitting residuals, defined as the difference between the raw and fitted data, are plotted as a function of delay time (ps). The small amplitude of these residuals, remaining within ± 0.01, indicates good fit quality. (b) *γ*_EEA_*n*(0) as a function of defect density, extracted from different fitting time windows. Error bars indicate deviations across fitting windows. (c) *k*_nr_^*A*^ extracted from different fitting time windows as a function of defect density. A linear trend is observed, with the grey shaded area representing the range of linear fits obtained from different fitting windows. Black dots indicate the average *k*_nr_^*A*^ values. The red line corresponds to a trace replotted using the average *γ*_DT_ and *k*′ values from all linear fits.

Following the analysis at *t* = 0 ps, we turn to the time-resolved evolution of the A-exciton signal. A temporal blueshift of the peak energy is observed across all samples, necessitating dynamic spectral corrections at each delay step. To accurately extract decay kinetics, a time-dependent fitting procedure was employed: a spectral window was adaptively centred on the A-exciton resonance, and the peak was modeled using asymmetric Gaussian functions to determine both position and amplitude (Fig. S3). The resulting normalized intensity traces are plotted on a logarithmic scale in Fig. 5(a). A clear trend emerges: higher defect densities yield faster exciton decay. As discussed in the steady-state analysis, two ultrafast nonradiative channels dominate: EEA and DT, schematically illustrated in Fig. 3(a). Notably, EEA is activated only at sufficiently high initial exciton densities. To assess this, the initial exciton density *n*(0) for each sample was estimated using the relation:^46^5
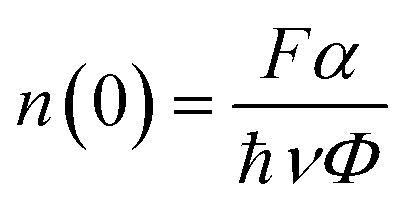


Here, *F* denotes the pump fluence (J cm^−2^), *α* the absorbance at the pump wavelength (unitless), ħ*ν* the photon energy, and *Φ* the exciton generation efficiency. Assuming an exciton generation yield of unity and an absorbance of 0.06 at 400 nm for the monolayer MOCVD-grown WS_2_ sample,^43,47^ the initial exciton density is estimated to be 1.606 × 10^13^ cm^−2^ for a pump fluence of 0.133 mJ cm^−2^. Despite this high density, no signs of exciton generation saturation are observed (Fig. S8).

Given the high *n*(0), EEA must be considered alongside DT in the decay kinetics. To account for both pathways, we apply a rate equation model that includes EEA, DT, and radiative recombination as parallel, competing channels:^48,49^6
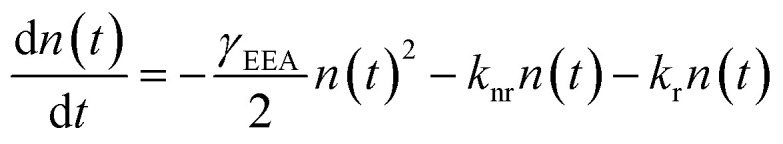


As previously discussed, the quantum efficiency is low (≈3.2 × 10^−5^) for the pristine sample, justifying the omission of the radiative recombination term in the decay model. EEA is treated as a second-order Auger-type process, in which one exciton recombines nonradiatively by transferring its energy to a second exciton, promoting it to a higher-energy state (Fig. 3(a)).^48,50^ In contrast, DT is modelled as a first-order process governed by the defect density and contributes to the effective nonradiative rate constant *k*_nr_, alongside other ultrafast decay pathways, which will be discussed in detail later. The competing nature of these channels motivates the following kinetic model:7
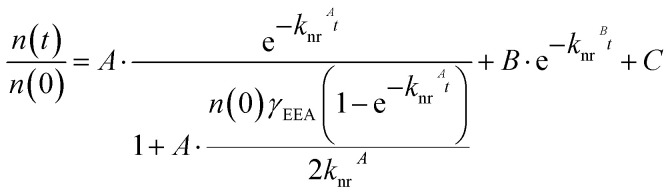


Eqn (7) captures the exciton decay dynamics using two distinct time-dependent components. The first term, scaled by amplitude *A*, is derived from the solution of a model considering the EEA channel,^48^ with effective nonradiative decay rate *k*_nr_^*A*^. The parameter *γ*_EEA_ is the EEA rate coefficient. The second term, weighted with amplitude *B*, accounts for slower, first-order nonradiative processes, such as exciton diffusion to grain boundaries.^51^ This dominates at later times when EEA becomes negligible, with rate constant *k*_nr_^*B*^. The separation of the two terms suggests that EEA is active only within the first few picoseconds. The constant *C* accounts for a long-lived signal offset outside the experimental detection window.

Prior to decay fitting, the instrument response function (IRF) was determined from the leading edge of the signal, including the negative delay region, yielding *σ*_IRF_ ≈ 65 fs. This is comparable to the pump pulse duration, with a full width at half maximum (FWHM) ≈ 100–150 fs (Fig. S9). To avoid influence from the initial rise, the fit range was restricted to times *t* ≥ 0.3 ps, with the upper limit varied between 25 and 50 ps in 0.5 ps steps to assess sensitivity to the fitting window. Fig. 5(a) shows an example for the sample with the highest defect density. The extracted values for *γ*_EEA_*n*(0) and *k*_nr_^*A*^ vary systematically with defect density (Fig. 5(b and c)). Error bars in grey represent the spread of values across the different fitting windows, illustrating the sensitivity of the extracted parameters to the chosen temporal range.

Starting from the EEA component, we extract the annihilation rate coefficient *γ*_EEA_ using the estimated initial exciton density *n*(0) = 1.606 × 10^13^ cm^−2^. This yields *γ*_EEA_ values ranging from approximately 0.05 to 0.14 cm^2^ s^−1^, in good agreement with previous reports.^4,8,50,52,53^ Notably, *γ*_EEA_*n*(0) becomes difficult to extract from the fitting (approaches zero) at the highest defect density (Fig. 5(b)). This suggests a critical threshold in the defect-to-exciton ratio of ≈ 3.5, below which two excitons can still interact before encountering a defect, whereas above this value, DT dominates the decay dynamics.

For *k*_nr_^*A*^, a similar assumption to that used in the steady-state PL analysis (eqn (2) and (3)) is applied. However, unlike in PL, the high initial exciton densities in TA experiments can lead to partial saturation of defect states, since their recovery upon occupation occurs on nanosecond timescales.^12^ As a result, excess excitons may decay *via* alternative nonradiative channels. These additional pathways can be ultrafast, such as *k*′, which may involve trapping at intrinsic defect species not modified by irradiation. Moreover, carrier–phonon scattering may contribute,^43,54,55^ particularly under strong excitation. Rapid relaxation of high-energy excitons generates a significant phonon population, which may subsequently scatter with excitons at the bandedge.

The contribution of additional ultrafast channels, denoted as *k*′, can be extracted by analyzing the linear dependence of *k*_nr_^*A*^ on defect density. The corresponding fit results are shown in Fig. 5(c), with shaded regions representing variations due to the choice of fitting window. A representative linear fit (red line) is plotted using the average values of *k*′ ≈ 0.55 ps^−1^ and *γ*_DT_ = 2.15 × 10^−14^ cm^2^ ps^−1^. Notably, when comparing the *γ*_DT_ values obtained from steady-state and time-resolved measurements—3.0 × 10^−13^ cm^2^ ps^−1^*vs.* 2.15 × 10^−14^ cm^2^ ps^−1^, respectively—a clear discrepancy of more than an order of magnitude emerges. This result suggests that at high initial exciton densities, excitons may either undergo annihilation before reaching a defect site or encounter a defect that is already occupied by a previously trapped exciton, thereby preventing further nonradiative recombination on ultrafast timescales. Consequently, the effective defect trapping rate per unit defect decreases with increasing exciton density. In line with this, the extracted *k*_DT_ from time-resolved measurements is significantly lower than the steady-state value, supporting our assumption that *k*′ can be neglected in steady-state analyses but should be retained in the TA model.

### 2.5 Diffusion-limited competition

To further examine the interplay between EEA and DT, we consider that both processes are governed by diffusion-limited kinetics. In the case of EEA, excitons undergo random diffusion until they encounter another exciton within a characteristic annihilation radius *R*_EEA_, leading to immediate recombination.^56^ According to Smoluchowski's theory, *γ*_EEA_ is proportional to the exciton diffusion coefficient *D* for *L*_D_ ≫ *R*_EEA_, where 
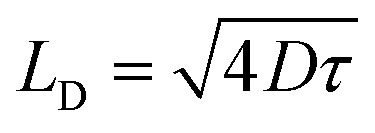
 denotes the exciton diffusion length^56–59^ and *τ* is the effective exciton lifetime. We propose a simplified model in which DT follows an analogous mechanism: an exciton is captured once it diffuses within a trapping radius *R*_DT_ of a defect.

To illustrate the diffusion-limited character of both decay pathways, we compare representative exciton lifetimes and diffusion lengths under EEA- and DT-dominated conditions. For simplicity, we incorporate the additional nonradiative term *k*′ into the DT channel, treating it as one single competing channel to EEA. We begin with a scenario where both the initial exciton density and the defect density are approximately 1.6 × 10^13^ cm^−2^, corresponding to an average spacing of ≈2.5 nm between excitons or defects. Under EEA-limited conditions, the effective exciton lifetime is 0.7 ps, while in the DT-limited case, it is 1.3 ps. Assuming a diffusion coefficient of *D* = 0.3 cm^2^ s^−1^,^60^ this yields diffusion lengths of ≃9 nm (EEA) and ≃13 nm (DT), respectively.

Given that both estimates refer to the same average spatial separation, the comparable diffusion lengths suggest that the effective annihilation and trapping radii are of similar magnitude. At the highest defect density, where the exciton lifetime shortens to 0.6 ps, the diffusion length falls below that of the EEA regime. This indicates that DT becomes the dominant process, effectively suppressing the EEA channel. Consistent with this interpretation, the extracted value of *γ*_EEA_*n*(0) approaches zero in the most defective sample.

### 2.6 Conclusions

In this work, we quantitatively investigated exciton decay pathways in wafer-scale MOCVD-grown monolayer WS_2_ by combining steady-state quantum efficiency measurements and femtosecond transient absorption spectroscopy. Controlled defect introduction enabled the estimation of intrinsic sulfur vacancy densities and unit defect trapping rates. Time-resolved measurements provided strong evidence for occupied in-gap defect states and reveal a competition between exciton–exciton annihilation and defect trapping that depends on both excitation conditions and defect concentrations.

A critical defect-to-exciton ratio (≈3.5) was identified as the threshold for activating EEA, beyond which DT dominates due to shorter exciton diffusion lengths. Under high exciton densities, partial defect saturation reduces trapping efficiency, further modulating the recombination landscape.

These findings establish a quantitative framework for diffusion-limited exciton dynamics, providing critical guidance for future defect engineering strategies aimed at enhancing the functionality of device applications based on large-area two-dimensional TMDCs.

## 3 Materials and methods

### 3.1 MOCVD growth

The MOCVD WS_2_ sample was synthesized in a commercial AIXTRON CCS multi-wafer MOCVD reactor. Tungsten hexacarbonyl (W(CO)_6_) and ditert-butyl sulfide (DTBS) were used as precursors. H_2_ was chosen as carrier gas. The deposition of WS_2_ includes two stages: the nucleation process (stage I) at 750 °C for 10 min and lateral growth (stage II) at 800 °C for 95 min with reduced W(CO)_6_ flux. The substrate is double-side polished *c*-plane sapphire with a 0.2° off-cut towards the m-plane.

### 3.2 Ion bombardment

For defect creation, the ion sputtering gun model IG35/IG70 from OCI Vacuum Microengineering Inc. was used. Argon gas with 99.999% purity from Air Liquide was filled into the chamber until a pressure 5 × 10^−6^ mbar was reached. The current density was set to 0.088 µA cm^−2^ corresponding to an ion flux of 5.5 × 10^11^ ions per cm^2^ s. The samples were exposed to the ion beam until the targeted fluence was reached.

### 3.3 SEM

SEM measurements were performed with a Zeiss Sigma HV, with a voltage of 10 kV, at a working distance of 2.6 mm, an aperture of 20 µm and under a stage tilt of 0.4°, enabling a magnification of 60 000.

### 3.4 PL and Raman spectroscopy

All PL and Raman measurements were performed with a Witec Alpha300 R setup, using a 532 nm cw laser. The laser spot diameter is estimated to be around 590 nm, which results in a laser power of 0.36 kW cm^−2^. The exciton formation rate is estimated to be approximately 7 × 10^10^ ps^−1^ cm^−2^.

### 3.5 QE determination

To determine the *QE*, the relative determination method was used. CdSe quantum dots spin-coated onto a sapphire substrate were used as a reference sample. The internal quantum efficiency of the reference sample was determined by an integrating sphere method and was obtained as 66%. Further, the absorption of the MOCVD sample and the reference sample at 532 nm excitation wavelength was measured as 7% and 8% respectively, enabling the determination of the *QE* through PL measurements.

### 3.6 Transient absorption spectroscopy

Femtosecond broadband transient absorption measurements were performed using a pump–probe system driven by a Pharos-SP laser (Light Conversion). The laser delivers fundamental pulses with a central wavelength of 1030 nm, a pulse duration of 170 fs, and an energy of 1 mJ, operating at a 6 kHz repetition rate. The pump pulses were generated using an optical parametric amplifier (Orpheus) with a second harmonic generation (SHG) module. Under 400 nm and 700 nm pumps, the samples were excited with fluences of 0.133 mJ cm^−2^ and 2.541 mJ cm^−2^. The probe beam consisted of a white-light supercontinuum, produced by focusing a small fraction of the fundamental beam onto a YAG crystal, yielding a probed wavelength range of 500–950 nm (corresponding to an energy range of 1.3–2.4 eV). The pump and probe spot sizes are 250 µm and 80 µm, respectively. They are linearly polarized and oriented perpendicular to each other. The arrival time of the probe pulses relative to the pump pulses was precisely controlled using an optical delay stage. Multiple scans were run and averaged for data analysis, and to rule out any alteration of the sample through the lasers, each scan in a row was checked for changes.

### 3.7 DFT calculations

First-principles calculations were performed using density functional theory (DFT) with VASP 6.3.0 version package.^61,62^ The interactions between ions and valence electrons were described using the projector-augmented wave (PAW)^63^ method, and electronic exchange and correlation were treated by the generalized gradient approximation of Perdew, Burke, and Ernzerhof (PBE).^64^ We employed a plane wave cut-off energy of 500 eV. The material models, including the defective system, were built in a 6 × 6 × 1 supercell with 15 Å vacuum to avoid interaction with the periodic images, which corresponds to a defect density of 2.1 × 10^13^ cm^−2^. A 9 × 9 × 1 Monkhorst–Pack *k*-point grid was used to sample the 2D Brillouin zone.

## Author contributions

R. Z., L. D., G. S., and M. S. jointly conceived the idea of the study. R. Z., under the supervision of G. S., and L. D., under the supervision of M. S., co-wrote the main content of the manuscript and contributed equally to the work. R. Z. performed the transient absorption measurements and contributed to data analysis and discussion. L. D. prepared the samples, conducted ion bombardment, performed Raman and steady-state quantum efficiency measurements and contributed to data analysis and discussion. D. S., supervised by P. K., performed the density functional theory (DFT) calculations and participated in the discussion. C. V., under the supervision of G. S., assisted with TA data analysis and contributed to the discussion. Y. D., under the supervision of A. V. and H. K., provided the pristine MOCVD-grown samples and performed scanning electron microscopy (SEM) measurements. M. H. from Aixtron SE contributed to the coordination of the sample preparation project using a commercial MOCVD tool. T. F., under the supervision of G. B., performed quantum efficiency measurements on the pristine MOCVD samples. All authors contributed to the interpretation of the results and to the preparation of the manuscript. G. S. and M. S. supervised the overall project.

## Conflicts of interest

The authors declare no conflict of interest.

## Supplementary Material

SC-OLF-D5SC07343J-s001

SC-OLF-D5SC07343J-s002

## Data Availability

The data supporting this article have been included as part of the supplementary information (SI) and are available *via* the University of Waterloo Dataverse (Borealis) at https://doi.org/10.5683/SP3/ZDPLRA. Supplementary information: trion and exciton fitting of photoluminescence spectra; colourmaps of transient absorption spectra for other samples; asymmetric Gaussian fitting of the A-exciton peak; simulated STM and electronic structure of charged sulfur vacancies; defect-dependent shifts of the A-exciton peak under 400 nm and 700 nm excitation; time-resolved blue shifts and fitting of their rate constants; analysis of exciton generation saturation at high pump fluence; and instrument response function analysis of ultrafast exciton dynamics. See DOI: https://doi.org/10.1039/d5sc07343j.
