# Supplementary material for: Defect-engineered competition between exciton annihilation and trapping in MOCVD WS2
Source: Chem Sci. 2025 Nov 14;17(2):1176–85. doi: 10.1039/d5sc07343j (PMC12631797; doi:10.1039/d5sc07343j)
Supplement: SC-017-D5SC07343J-s001 [file SC-017-D5SC07343J-s001.pdf]

Supplementary Information (SI) for Chemical Science.
This journal is © The Royal Society of Chemistry 2025

Electronic Supplementary Material

Defect-Engineered Competition Between Exciton Annihilation and Trapping in MOCVD WS_2

Ruofei Zheng[a,†], Leon Daniel[b,†], Dedi Sutarma[b], Christian Viernes[a], Yingfang Ding[c], Tobiloba Fabunmi[d], Gerd Bacher[d], Michael Heuken[e], Holger Kalisch[c], Andrei Vescan[c], Peter Kratzer[b], Marika Schleberger[b,*], Germán Sciaini[a,*]

[a]*Department of Chemistry, University of Waterloo, Waterloo, Ontario N2L 3G1, Canada*
[b]*Fakultät für Physik and CENIDE, University of Duisburg-Essen, Duisburg 47057, Germany*
[c]*Compound Semiconductor Technology, RWTH Aachen University, Aachen 52074, Germany*
[d]*Werkstoffe der Elektrotechnik and CENIDE, University of Duisburg-Essen, Duisburg 47057, Germany*
[e]*AIXTRON SE, Herzogenrath 52134, Germany*

† These authors contributed equally.
* Correspondence: marika.schleberger@uni-due.de, gsciaini@uwaterloo.ca

1 Trion and Exciton Fitting

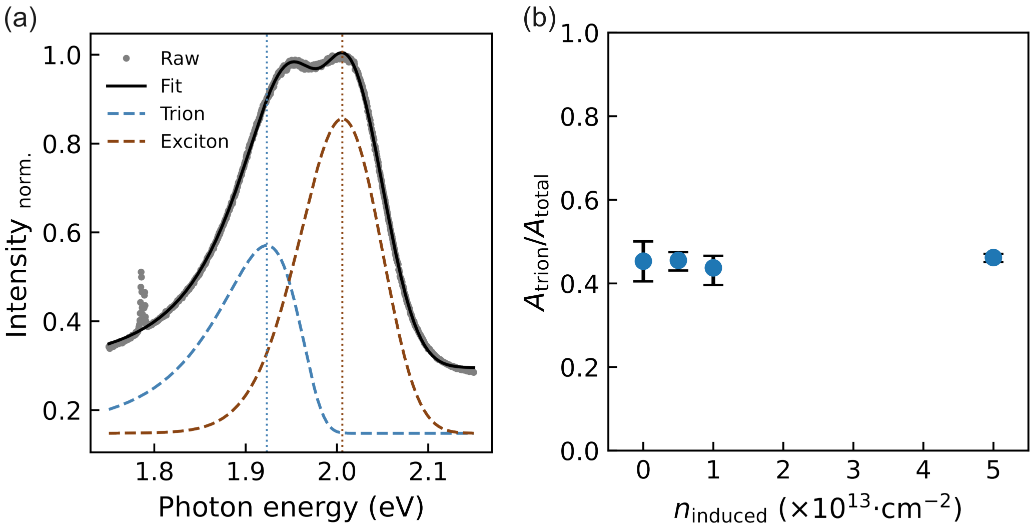

Fig. S1 (a) Exemplary trion and exciton spectral fits for the pristine sample. (b) Trion-to-total integrated area ratio for pristine and irradiated samples with different induced defect densities.

For each sample with a different induced defect density, five PL spectra were collected and fitted using two asymmetric Gaussian functions to achieve better fitting accuracy. The details of this procedure are discussed in the following section, *Asymmetric Gaussian Function Fitting*. The two fitted peaks were identified as emissions from the trion and exciton, respectively[1]. The integrated areas of the trion and the total emission were then calculated, revealing an overall similar trion-to-total ratio across the various samples.

2 Colormaps of Other Samples

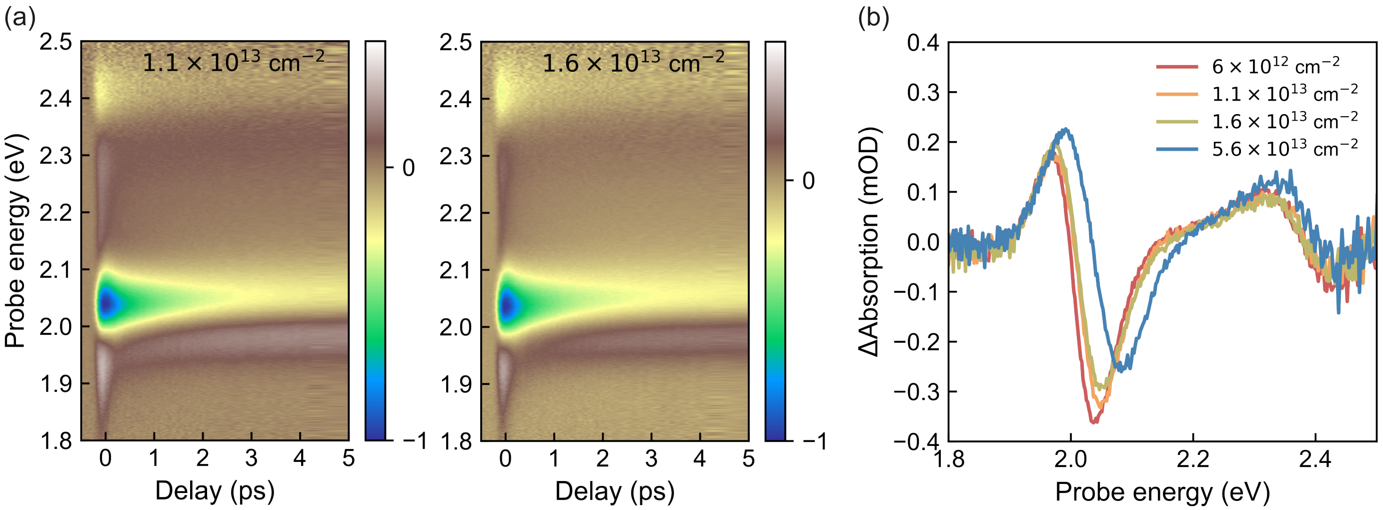

Fig. S2 (a) Colormaps of the TA spectra for the samples with defect densities of 1.1×10^{13} cm^{-2} (left) and 1.6×10^{13} cm^{-2} (right). The normalized differential absorption intensity is plotted as a function of probe energy (eV) and pump-probe delay (ps). (b) Comparison of spectra at 2 ps across the four samples.

3 Asymmetric Gaussian Function Fitting

An asymmetric Gaussian function is used to fit the A exciton Pauli blocking peak at each time delay, as described by the following equation[2]:

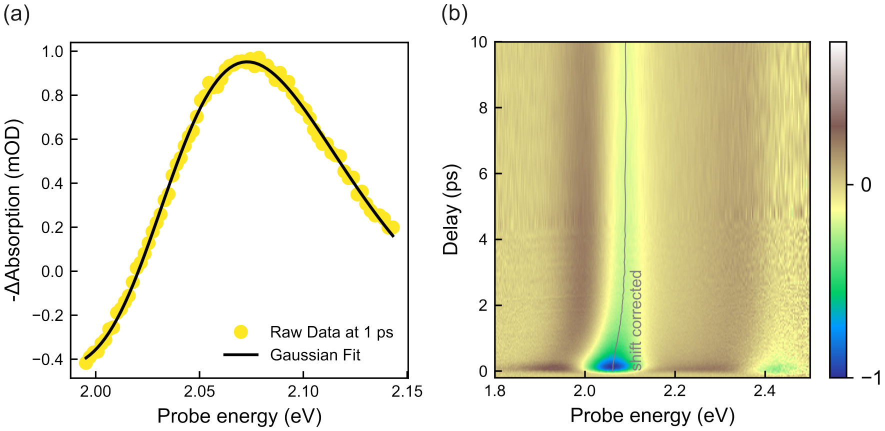

Fig. S3 (a) An example of asymmetric Gaussian function fitting for the A exciton peak; the spectrum is taken at 1 ps for the sample with a defect density of 5.6×10^{13} cm^{-2}. The extracted peak positions at each time step are replotted as a gray line in the colormap (b).

$$a\cdot\exp\left(\frac{-(x-x_0)^2}{2(\sigma+b(x-x_0))^2}\right)+c \tag{1}$$

where $\sigma+b(x-x_0)$ dynamically modifies the width of the Gaussian peak as a function of x, and b introduces asymmetry in the peak shape. The term c represents a baseline offset, shifting the entire function vertically. Additionally, due to the observed blue shift over time, the spectral window is adjusted dynamically to ensure proper selection of the A exciton peak at each time step. The center of the window is continuously redefined based on the previously fitted peak positions.

4 Charged Defect (V_S) of WS_2

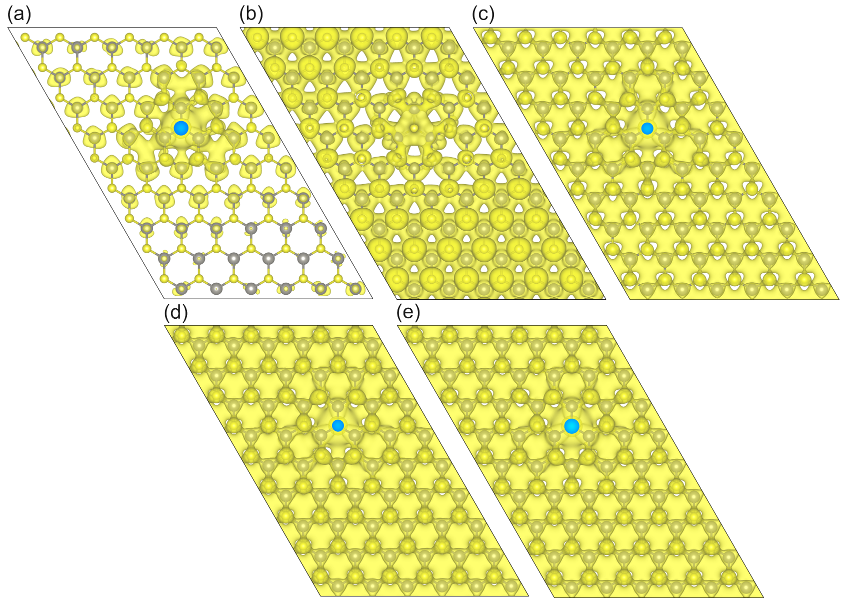

Fig. S4 Simulated STM images of WS_2 with a sulfur vacancy under different charge states: (a) −1, (b) −2, (c) 0, (d) +1, and (e) +2. The energy interval for band contribution is E_F ±0.5 eV to mimic bias voltage. An isosurface value of 0.002 e Bohr^{-3} is used consistently across all images. The yellow color indicates charge accumulation, while the blue color marks charge depletion. Among the cases shown, the −2 charged defect shows characteristics of a non-defective charge density landscape.

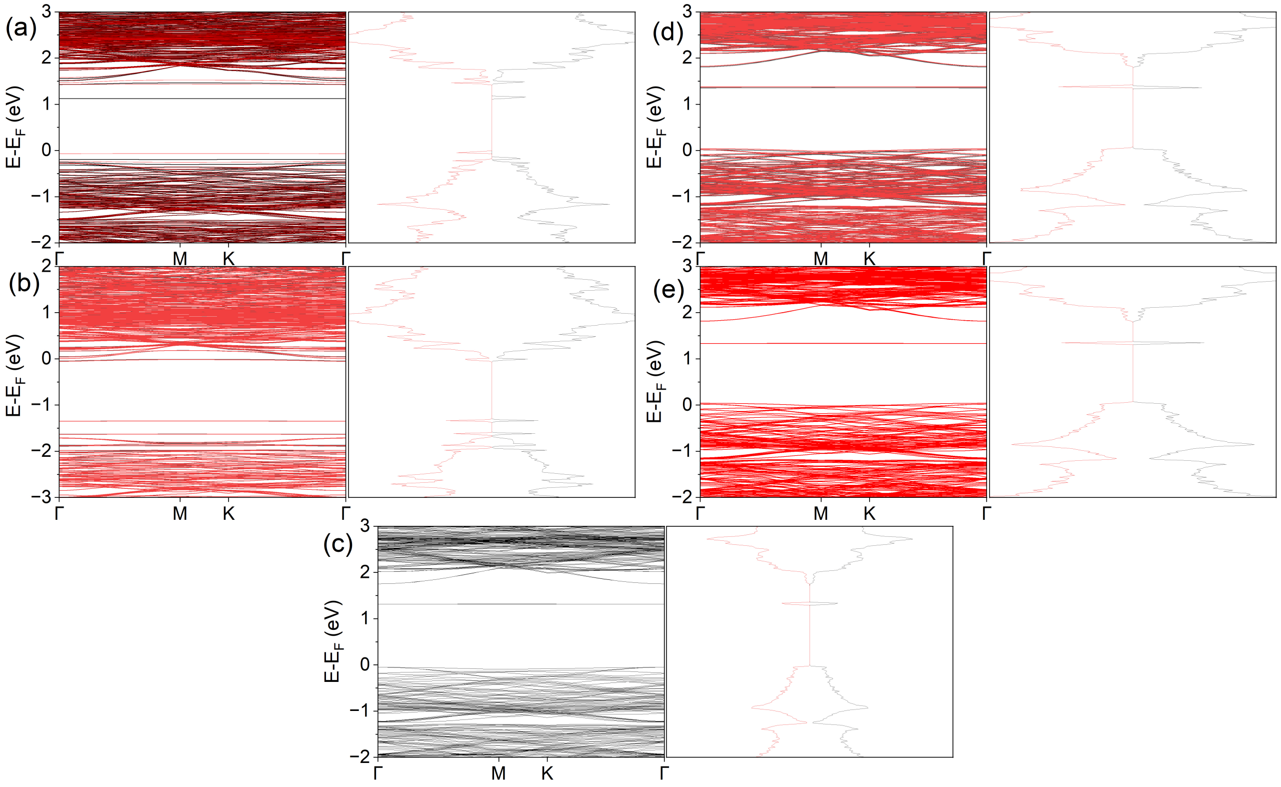

Fig. S5 Band Structure and Density of States (DOS) of WS_2 with a Sulfur Vacancy under different charge states: (a) −1, (b) −2, (c) 0, (d) +1, and (e) +2. The majority spin is shown in red. Positively charged defects introduce minimal changes compared to the neutral case. In contrast, negatively charged defects (−1 and −2) exhibit several new occupied states within the band gap.

5 A-Exciton Peak Position as a Function of Defect Density under 400 nm and 700 nm Excitation

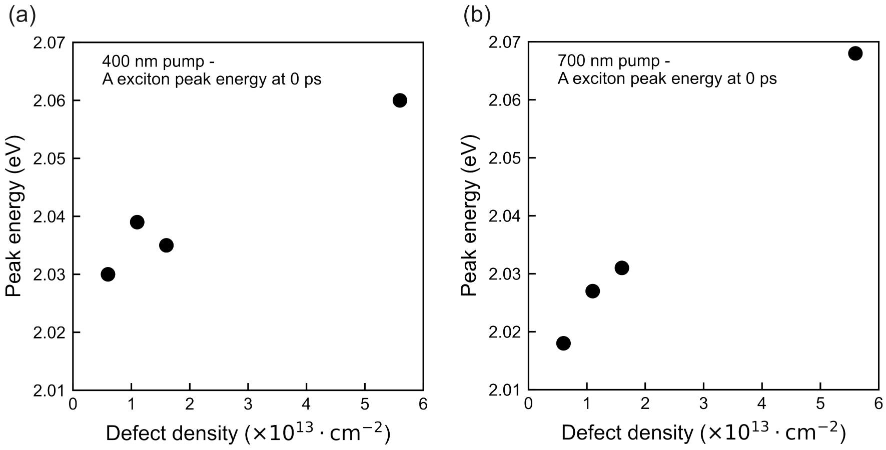

Fig. S6 Photon energy of the A exciton peak at 0 ps as a function of defect density under 400 nm pump excitation (a) and 700 nm pump excitation (b).

The A-exciton peak position at 0 ps, extracted from Gaussian fitting, exhibits a clear blue shift with increasing induced defect density.

6 Temporal Blue Shift of the A-Exciton Peak under 400 nm and 700 nm Pump Excitation

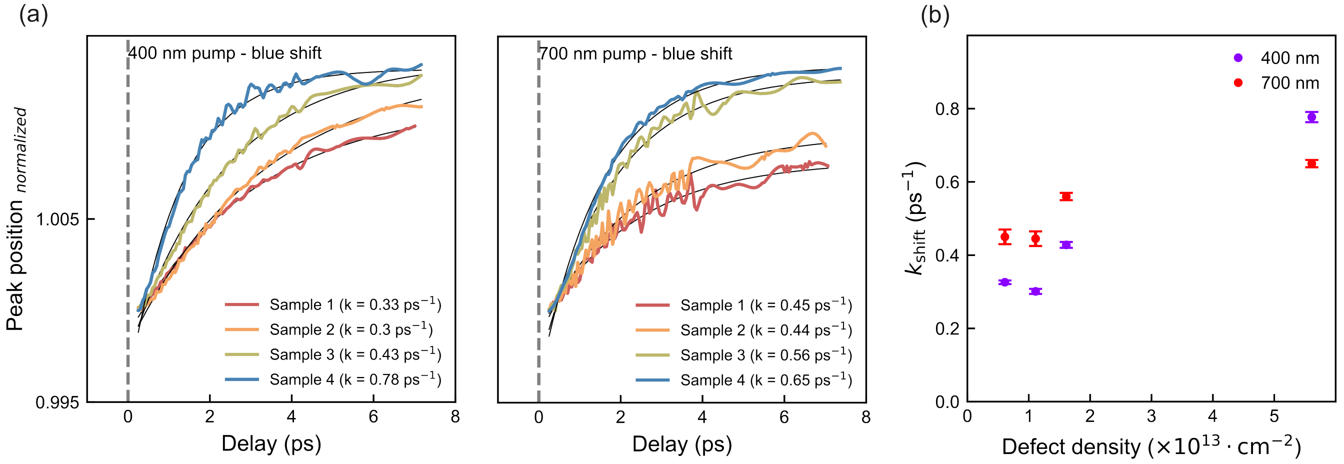

Fig. S7 (a) Time-dependent blue shift of the A-exciton peak under 400 nm (left) and 700 nm (right) pump excitation. The fitting results are shown as black traces, from which the blue-shift rate constant k is extracted. (b) Comparison of k across different samples, revealing an increasing trend with higher defect density.

One simple exponential rising function is applied to extract the blue shift rate:

$$1 - A \cdot e^{-k(t-t_0)} + c \tag{2}$$

To minimize the influence of initial perturbations and accurately characterize the shift trace, the first 0.25 ps is excluded from the analysis, and the fit is applied up to 7 ps. Samples 1 to 4 correspond to increasing induced defect density, from the lowest to the highest.

7 Exciton Generation Saturation

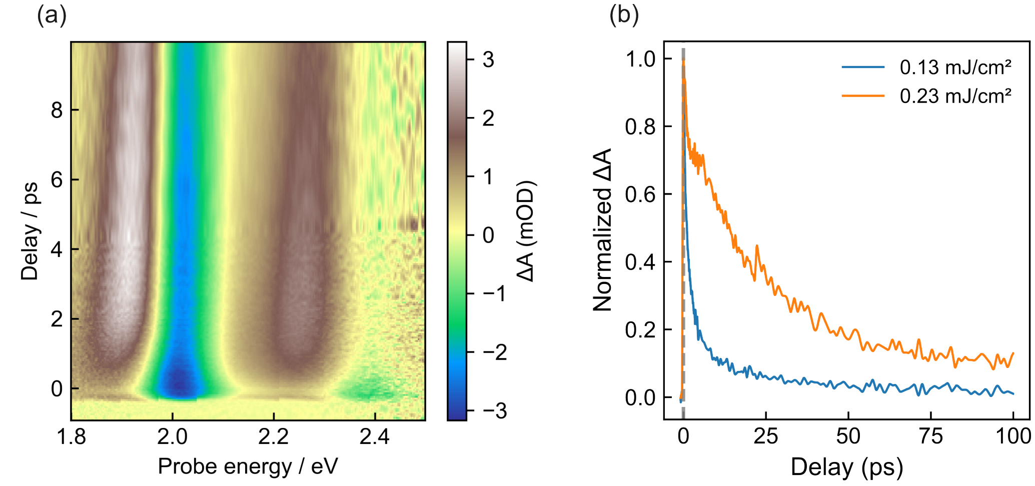

Fig. S8 (a) Colormap of the TA spectrum of the pristine sample at a specific pump fluence, where exciton generation tends to saturate, as indicated by the slow decay of the A exciton Pauli blocking signal. (b) Decay traces at the A exciton peak for the pristine sample under pump fluences of 0.13 mJ/cm^2 (blue) and 0.23 mJ/cm^2 (orange). At 0.23 mJ/cm^2, an extremely slow decay is observed.

Saturation of exciton generation can occur when the pump fluence becomes excessively high, leading to a significantly slower decay in the transient signal, either in the ultrafast or longer time regimes[3]. In our case, a higher pump fluence

was intentionally applied to observe the saturation behavior, whereas all measurements used for analysis were conducted within the linear response regime.

8 Instrument Response Function Fitting

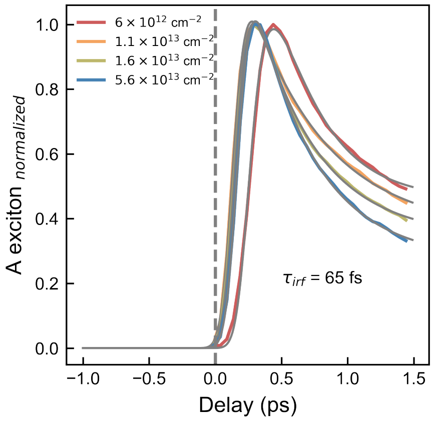

Fig. S9 (a) Normalized A exciton decay trace for each sample. The decay is fitted using the following function, yielding $\sigma_{\mathrm{irf}} = 65$ fs.

$$(H(t-t_0)(1-e^{-(t-t_0)/\tau_{\mathrm{rise}}})(Ae^{-(t-t_0)/\tau_1}+B)) * (e^{-\frac{1}{2}\cdot\frac{(t-t_0)^2}{\sigma_{\mathrm{irf}}^2}}) \tag{3}$$

To analyze the initial rise of the A exciton signal, corresponding to the generation of A excitons, a convolution of the instrument response function (IRF) with a Heaviside step function, multiplied by a rising and decaying exponential function, is applied. A-exciton formation involves the initial generation of a high-energy exciton continuum, followed by relaxation into lower-lying excitonic states. This relaxation process is typically faster than the instrument response[4]. For our samples, the average extracted instrument response time is $\sigma_{\mathrm{IRF}} \approx 65$ fs, which is comparable to the pump pulse duration, with a full width at half maximum (FWHM) of approximately 100–150 fs. The exciton generation time constant is $\tau_{\mathrm{rise}} \approx 55$ fs, except for the pristine sample, which shows a relatively longer $\tau_{\mathrm{rise}} \approx 100$ fs. These results indicate that defects may affect the exciton generation dynamics[4], which needs further investigation.

Notes and references

[1] J. W. Christopher, B. B. Goldberg and A. K. Swan, *Sci. Rep.*, 2017, **7**, 14062.

[2] G. A. Mohammed and M. Hou, *IEEE Trans. Biomed. Eng.*, 2016, **63**, 630–635.

[3] Y. Li, X. Wu, W. Liu, H. Xu and X. Liu, *Appl. Phys. Lett.*, 2021, **119**, 051106.

[4] C. Trovatello, F. Katsch, N. J. Borys, M. Selig, K. Yao, R. Borrego-Varillas, F. Scotognella, I. Kriegel, A. Yan, A. Zettl, P. J. Schuck, A. Knorr, G. Cerullo and S. D. Conte, *Nat. Commun.*, 2020, **11**, 5277.
