# Supplementary figures and images for: Defect-engineered competition between exciton annihilation and trapping in MOCVD WS2
Source: Chem Sci. 2025 Nov 14;17(2):1176–85. doi: 10.1039/d5sc07343j (PMC12631797; doi:10.1039/d5sc07343j)

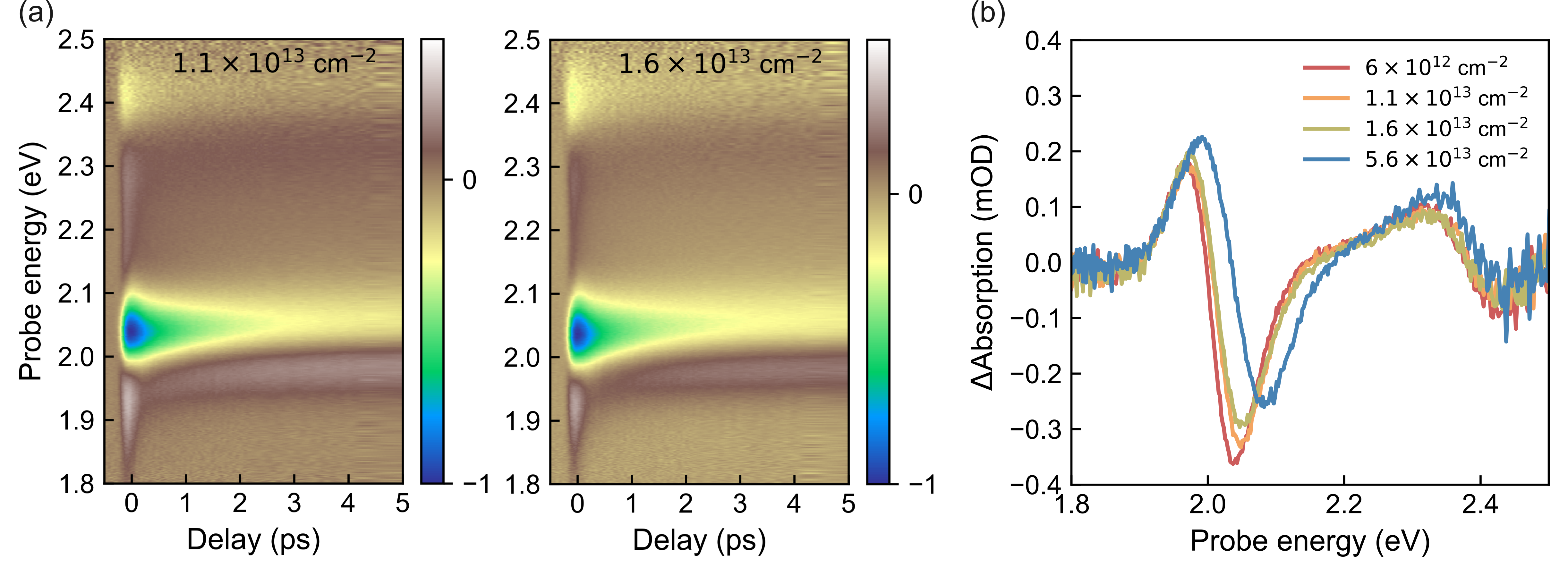

Supplement: SC-017-D5SC07343J-s002 [file SC-017-D5SC07343J-s002.zip › supFigures/Fig sTA.png]

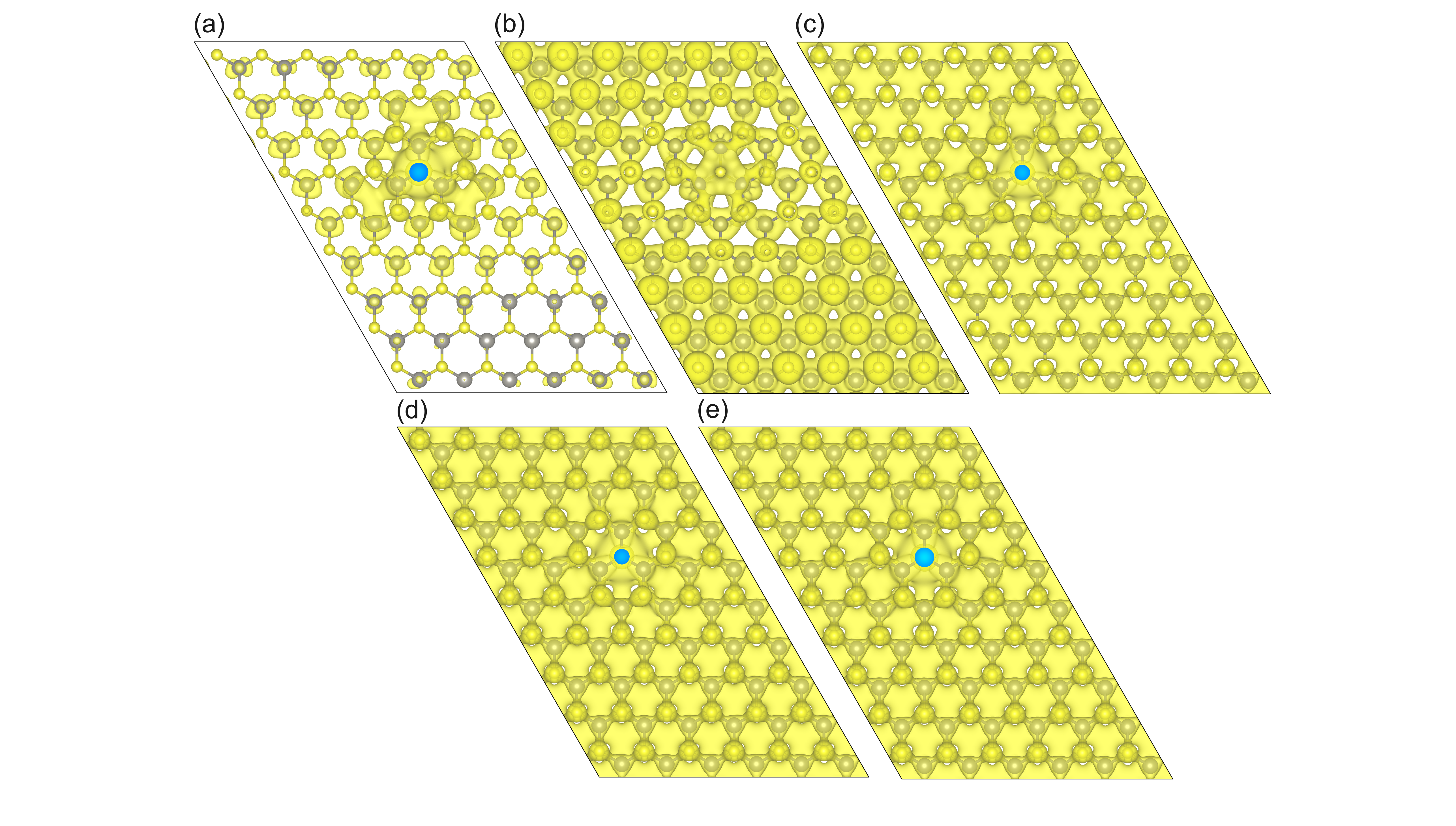

Supplement: SC-017-D5SC07343J-s002 [file SC-017-D5SC07343J-s002.zip › supFigures/Fig sSTM.png]

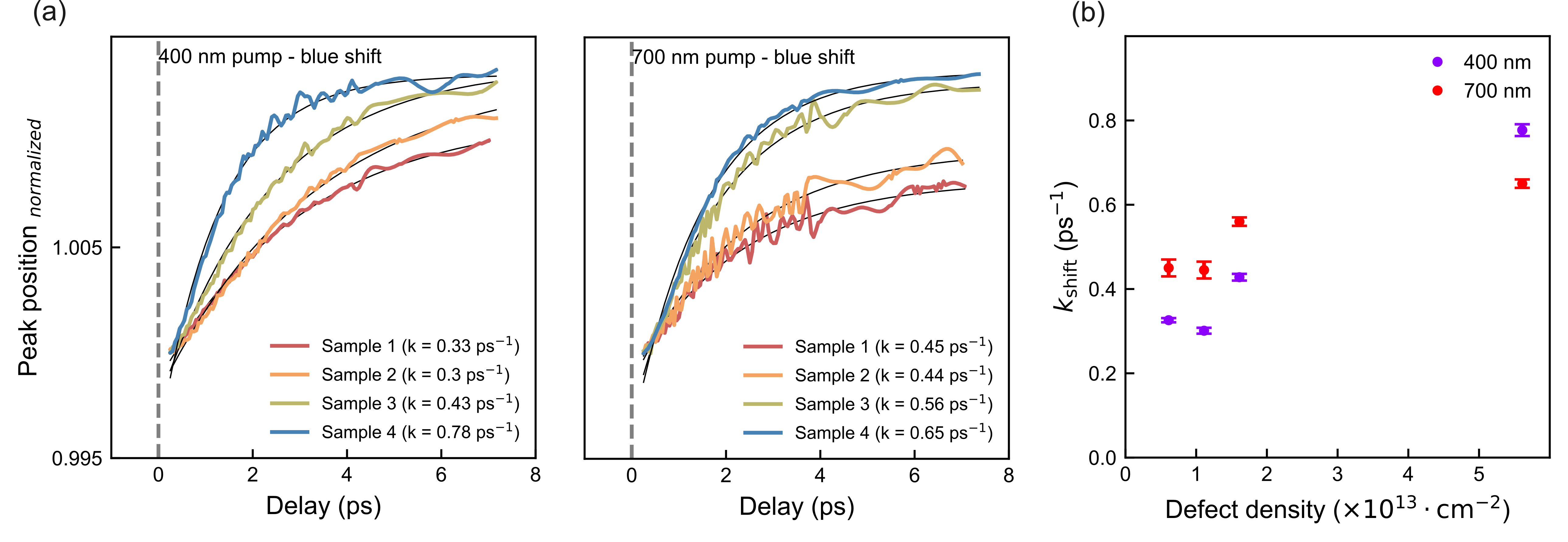

Supplement: SC-017-D5SC07343J-s002 [file SC-017-D5SC07343J-s002.zip › supFigures/Fig sPshift.png]

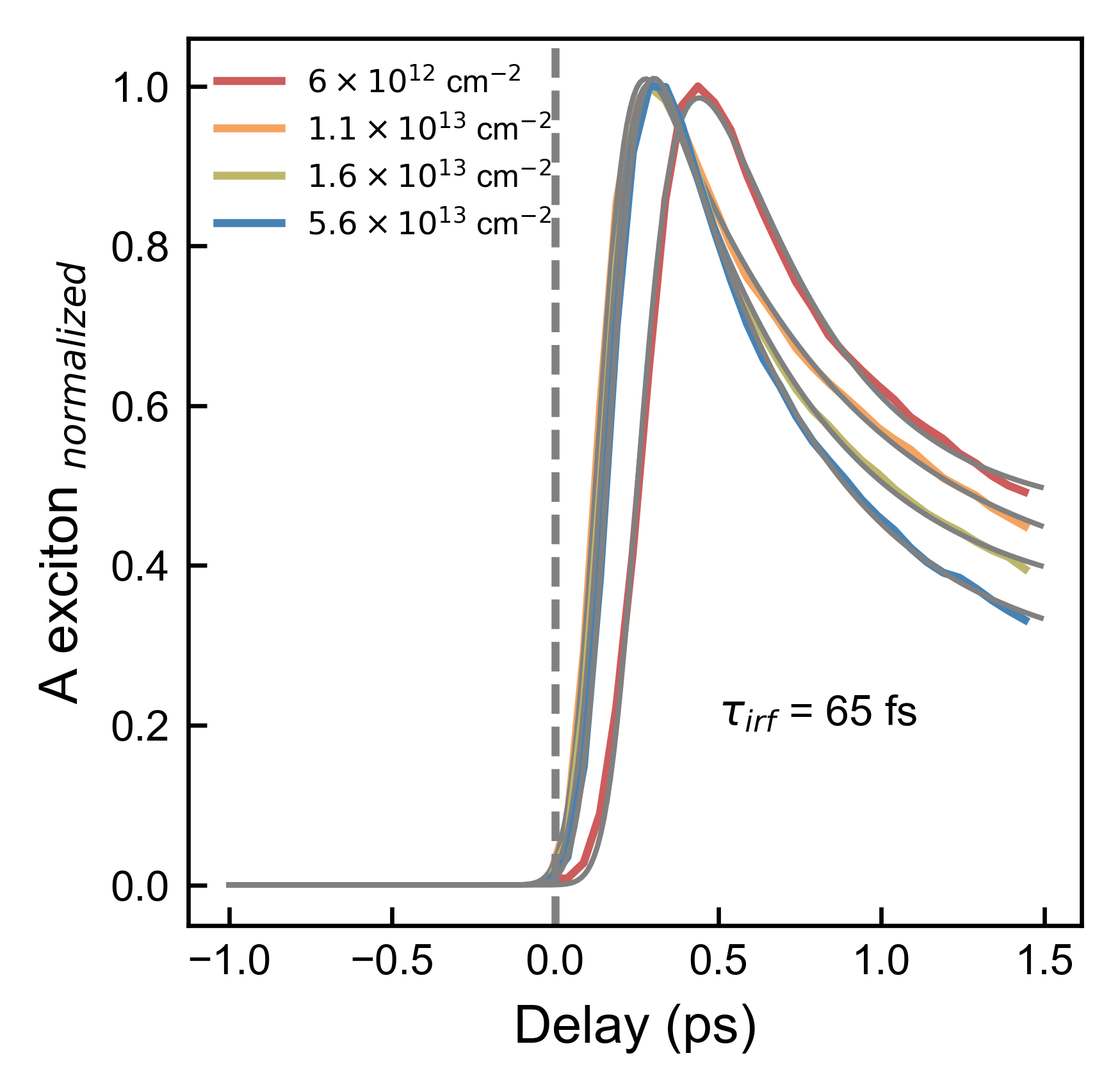

Supplement: SC-017-D5SC07343J-s002 [file SC-017-D5SC07343J-s002.zip › supFigures/Fig sIRF.png]

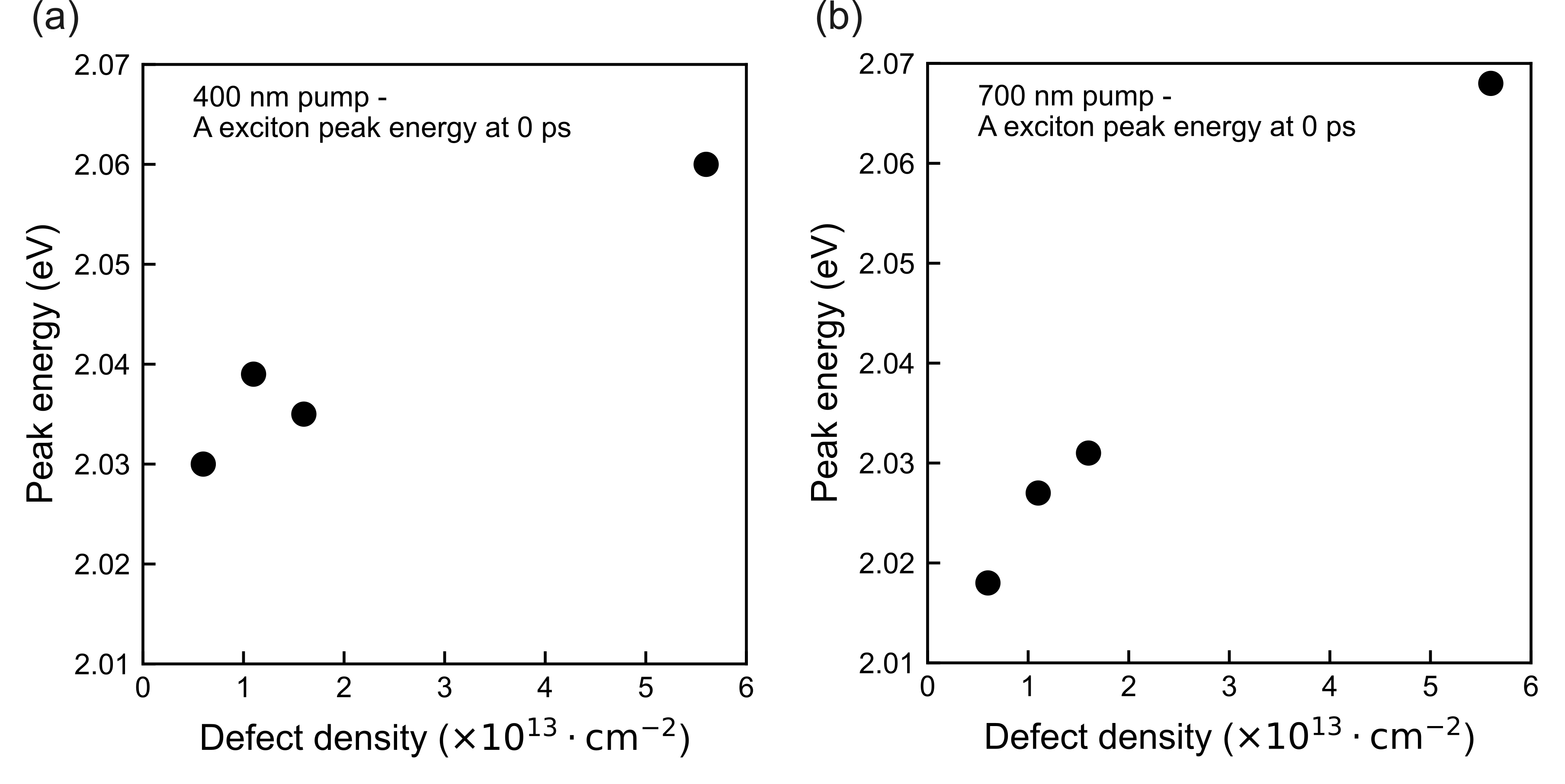

Supplement: SC-017-D5SC07343J-s002 [file SC-017-D5SC07343J-s002.zip › supFigures/Fig sPE.png]

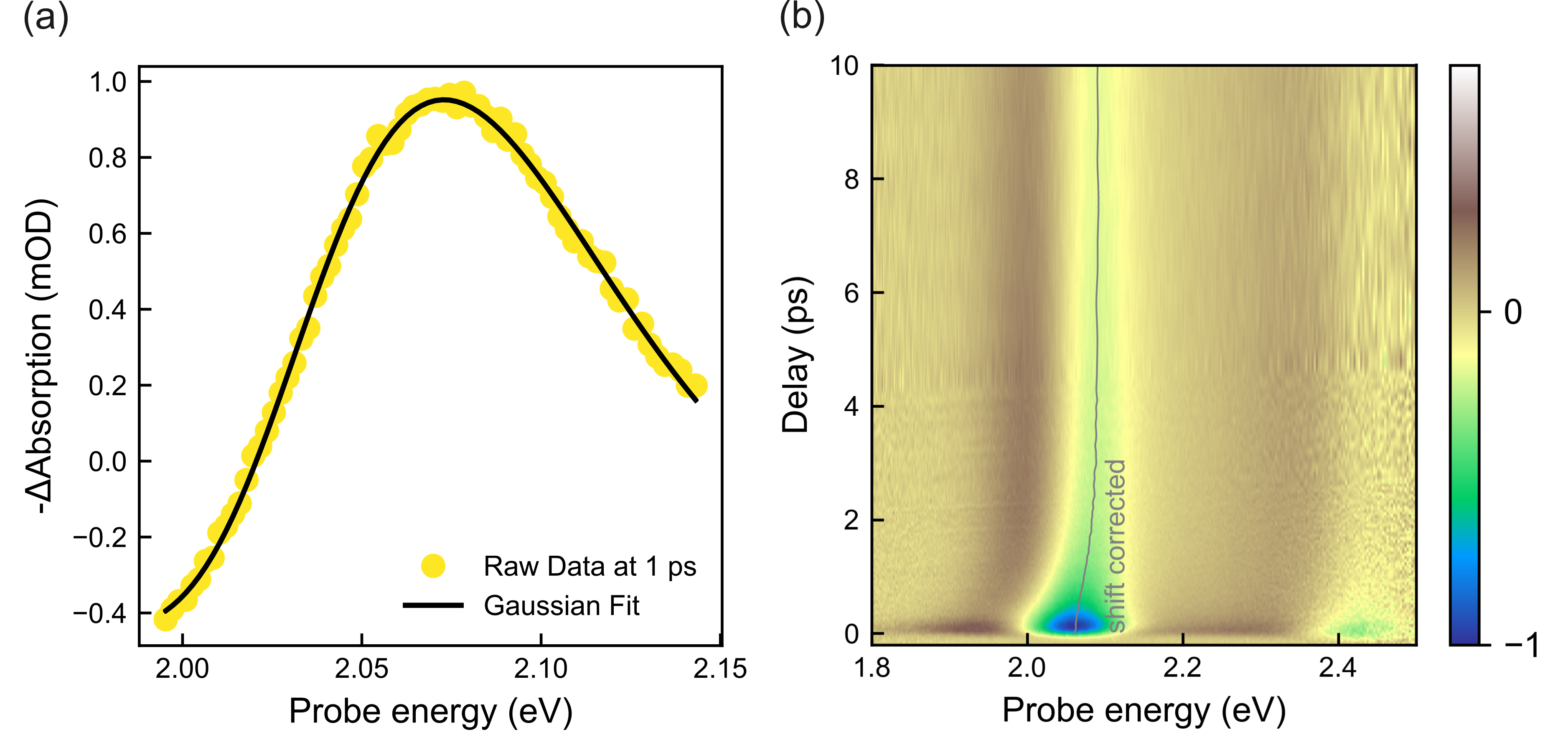

Supplement: SC-017-D5SC07343J-s002 [file SC-017-D5SC07343J-s002.zip › supFigures/Fig sGF.png]

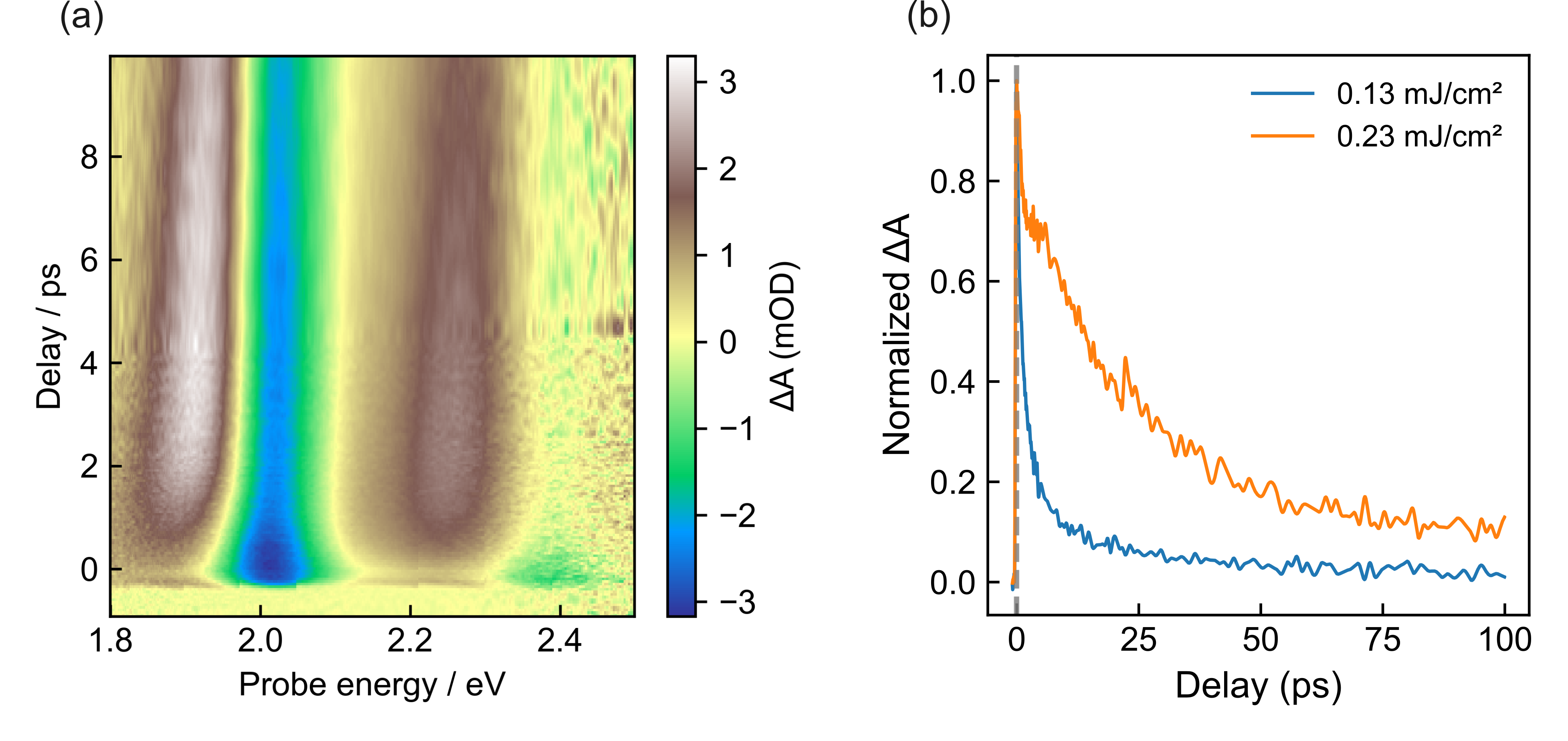

Supplement: SC-017-D5SC07343J-s002 [file SC-017-D5SC07343J-s002.zip › supFigures/Fig sFlu.png]

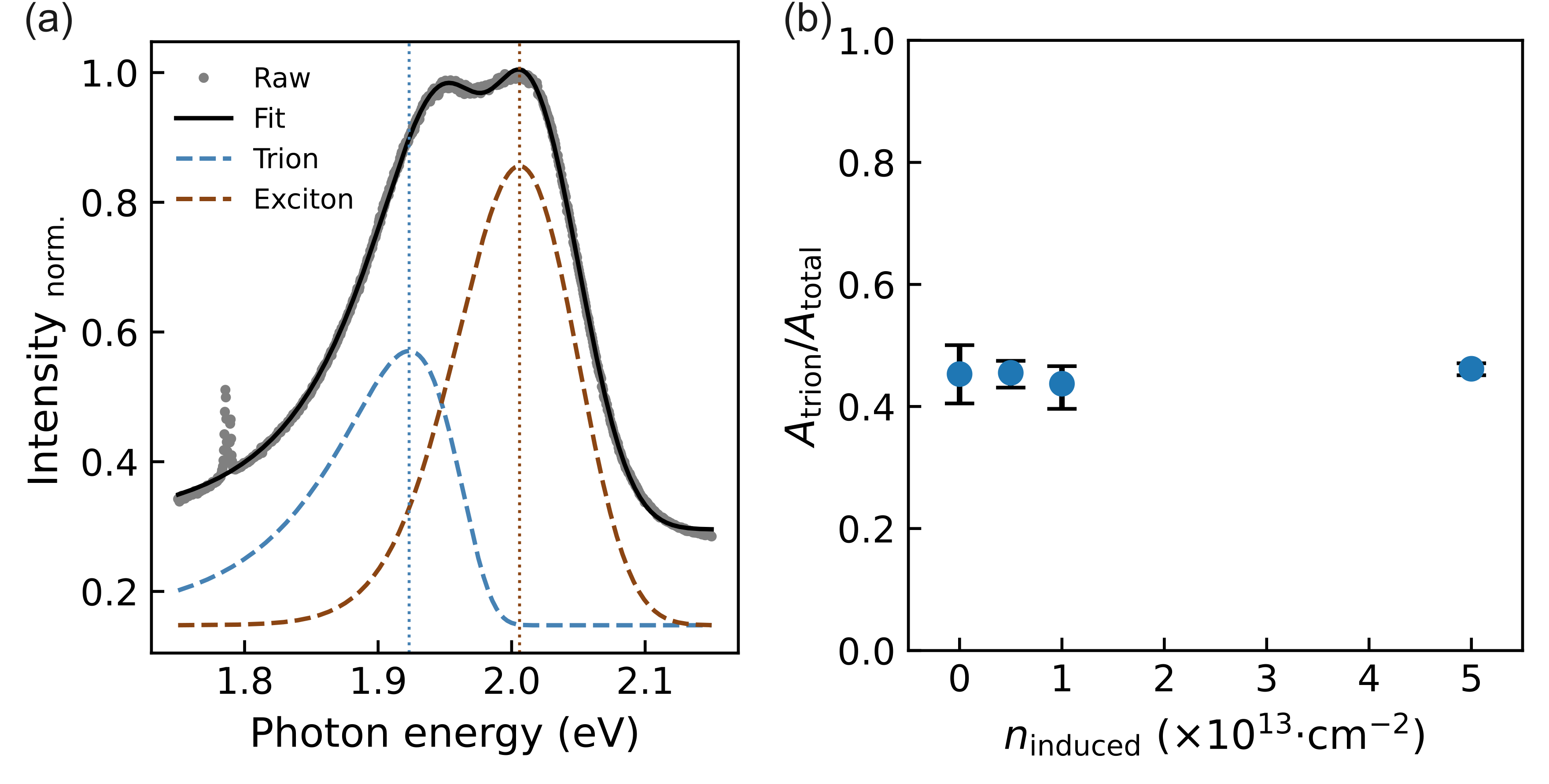

Supplement: SC-017-D5SC07343J-s002 [file SC-017-D5SC07343J-s002.zip › supFigures/Fig strion.png]

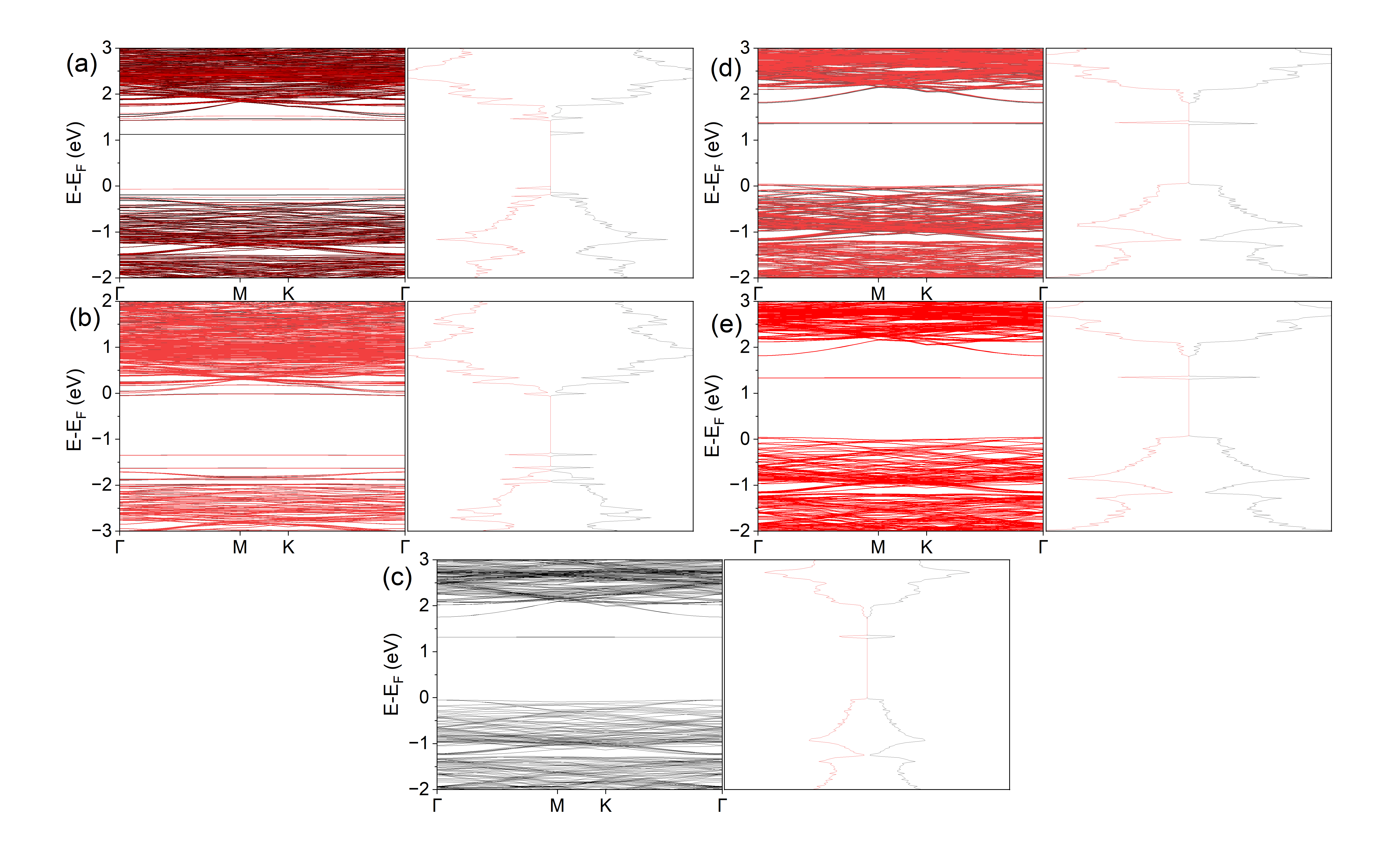

Supplement: SC-017-D5SC07343J-s002 [file SC-017-D5SC07343J-s002.zip › supFigures/charged.jpg]
